# Finger Prick to Finger Tip: Use of Mobile Phone Technology to Send PKU Blood Results

**DOI:** 10.1155/2018/2178346

**Published:** 2018-06-24

**Authors:** Anne Clark, Deirdre Deverell, Emma Corcoran, Margaret Macauley, Nicola Newcombe, Peter Branagan, Aoife Coughlan, Eimear Daly, Aoibhin Moore Heslin, Ellen Crushell, Joanne Hughes, Ina Knerr, Ahmad Monavari

**Affiliations:** ^1^National Centre for Inherited Metabolic Disorders, Temple Street Children's University Hospital, Dublin, Ireland; ^2^Paediatric Laboratory Medicine, Temple Street Children's University Hospital, Dublin, Ireland; ^3^Department of Information and Communication Technologies (ICT), Temple Street Children's University Hospital, Dublin, Ireland; ^4^Department of Research, Temple Street Children's University Hospital, Dublin, Ireland; ^5^University College Dublin, Belfield, Dublin, Ireland

## Abstract

The Metabolic Dietetic Team in the National Centre for Inherited Metabolic Disorders (NCIMD) in Ireland deals with approximately 120 weekly phenylalanine (Phe) levels for both adults and children. A review of 500 Phe levels highlighted that 52% of the results were within the target range. Collaboration between information and communication technologies (ICT) departments, metabolic laboratory, and metabolic dietitians enabled the development of the PKU texting system. Following a successful pilot study, the system was then offered to all PKU patients aged over 2 years. The Phe is analysed and authorised on the laboratory system. The demographics are matched with the patient mobile phone number. Text messages are then validated and sent by the dietitian via a web portal using the Defero SMS texting service. Approximately 290 patients/families currently use the texting system. In order to assess the effectiveness of this quality improvement initiative, a patient survey was carried out in 2017. This showed 87% rated the system as either very good or excellent. 94% agreed it was time saving. 84% felt there was no influence on dietary compliance. Analysis of financial implications on dietetic time over 21 months revealed savings of €3,275 and 580 hours of dietetic time. There is no evidence, two years after implementation, that the system has had an effect on either the Phe levels in terms of recommended range or frequency of sampling.

## 1. Introduction

Metabolic dietitians review approximately 120 phenylalanine (Phe) levels weekly and discuss these results with patients via telephone. A review carried out of 500 Phe levels in 2012 highlighted that 52% of the results were within the target range. This suggested that there were a large percentage of patients who did not require further follow-up by a dietitian on a regular basis.

There are approximately 500 patients both adults and children with phenylketonuria (PKU) attending the National Centre for Inherited Metabolic Disorders (NCIMD) in Temple Street Children's University Hospital. Patients with PKU require regular monitoring of their blood Phe levels. Regular monitoring of Phe levels in PKU patients is important as it is an indicator of compliance.

A pilot texting system was introduced from January 2015 to March 2015. The pilot period was 3 months. During this period, 28 parents received weekly text messages with the appropriate text messages for the Phe result via the Defero [1] texting service. Parents engaged with the dietitian as appropriate. Following this successful pilot period, patients with results within target range received a text with their/their child's Phe level directing them to continue with current management. Those with levels outside of the target range received a text advising them to contact the NCIMD for further advice and support.

Mobile phones are used by many people on daily basis. A survey completed by the Pew Research Center in 2012 surveyed 21 countries. 75% of those surveyed sent and accessed text messages. Mobile phone use was in fact most common in Kenya and Indonesia. These were two of the poorest nations who completed the survey [2]. 2016 was the first year that Ireland participated in Deloitte's Global Mobile Consumer Survey. 86% of Irish consumers either have access to or own a smartphone. 26% do not use their mobile phone to make phone calls on a regular basis. Usage is becoming more widespread using other channels such as texting. The 2016 survey highlighted that 28% of Irish consumers check text messages first after waking [3]. Jurecki et al. in 2017 studied adherence to clinic recommendations in PKU patients and demonstrated need for further improvement [4].

## 2. Aims and Objectives

The aim of this study was to examine the PKU results texting system in terms of patient acceptability, safety, and any financial implications. The objectives were to ensure that patients/parents received the correct advice via text in a timely matter without having a negative impact in terms of both metabolic control and the number of Phe samples posted for analysis.

## 3. Patients and Methods

### 3.1. Application Process

An application outlining the proposed project was submitted to the ICT manager for consideration. Following approval, a collaborative team was formed with representation from Dietetics, ICT, and Metabolic Laboratory to develop this novel texting system. The application process involved completion of a Project Initiation Document which was reviewed by both the Information and Communication Technologies Planning and Review and Strategy Committees. This business case was then sighed off by the hospital management board. This was necessary as it would impact on the ICT department. In addition hospital funding was required to develop the technology.

### 3.2. Data Protection Commission

In order to ensure patient confidentiality and safety compliance, the Data Protection Commission was consulted. Data are collected in line with the Data Protection Act 1988. In addition, it is in accordance with Temple Street Children's University Hospital Data Protection Policy.

### 3.3. Pilot Study

Commencing March 2015, a group of 27 patients and their guardians participated in a pilot study for three months. Assent was obtained from patients and consent from their parents where applicable. As Phe level results were generated by the laboratory system, they were checked firstly against a manual document, which is the method used to generate paper Phe results for the dietitian and on a webpage to ensure accuracy. There were no documented errors during this time. This meant that there was complete reliability and validity between the text messages sent to mobiles and the manual results generated from the laboratory system.

Following the pilot project, this system is offered to the eligible patient group (all patients over 2 years of age excluding maternal PKU) with no changes required from the pilot period.

### 3.4. Patients

The total number of patients involved in the pilot study was 27. The gender breakdown was 14 females and 13 males. The age range was 18 months to 4 years. The exclusion criteria were <2 years and pregnancy.

### 3.5. Routine Clinical Care in PKU Testing

Routine clinical care requires blood testing of all PKU patients. This is done by the patient at home. Drops of blood are spotted on to a sample card “dried blood spot” (DBS) by the patient or the patient's parent/guardian at home and posted to the Department of Paediatric Laboratory Medicine where the DBS Phe level is analysed. Prior to the pilot study, all results, normal or abnormal, were relayed to the patient by the dietitian by telephone.

Since the implementation of the texting system, once the test has been performed and authorised on the LIMS (Laboratory Information Management System), the result is automatically sent by the pathology server via a HL7 data interface. The interface server then matches the blood spot test results with the patient's details on a patient management system.

The interface server displays the blood Phe result along with the patient details and creates a web page viewable by the dietitian for validation who can then send a text message of the result to the patient or the patient's guardians. The mobile then receives a text message informing of their/their child's Phe result or to call the dietitian if the level is not within range.

### 3.6. Patient Survey

A survey was developed and circulated to the dietetic team. The signed off survey was then circulated to PKU patients/parents on our email database. The survey was available for completion for 3 weeks.

### 3.7. Statistical Analysis

Results were analysed using IBM SPSS Statistics 23.

## 4. Results

### 4.1. Financial Implications: 21 Months after Implementation

Table 1 highlights the financial savings which resulted from the implementation of the system. Text messages were costed at approximately €0.06 per text while the average phone call was estimated at €1.00. Prior to the implementation of the texting system, patients/parents contacted the dietitian for all levels, that is, both those within and out of range. Table 1 highlights savings of €3275 which is a result of the reduced cost of a text versus a phone call for levels within the recommended range. This then impacted on dietetic time as Table 2.

### 4.2. Impact on Dietetic Time: 21 Months after Implementation

Table 2 highlights the impact on dietetic time. The savings allowed the dietitian to spend more time with those patients who had levels out of range. Estimated phone call time per level averaged 10 minutes. Text messages averaged €0.06 per text. Phone calls averaged €0.10 per minute (10 minutes length of call = €1). This resulted in a saving of 580.5 hours.

### 4.3. Retrospective Review of the Mean Phe Level before and after Introduction (*µ*mol/l)

A retrospective review was carried out 12 months after implementation in order to assess whether or not the texting system has any impact on Phe levels. Median phenylalanine levels for the year before and after implementation of the texting system were collected for 212 patients. The number of samples before implementation was 97 levels and after implementation was 115 levels Table 3.

Cut-off points for the number of levels per year in each age group are determined as follows:

- 2 years–4 years: 48 levels per year
- 4.01 years–10 years: 24 levels per year
- 10.01+ years: 12 levels per year

The reference range used was 120–400 *µ*mol/l. Results were deemed significant at *p* ≤ 0.05. No significant difference was found before and after texting in the 2–4 years and 10+ years' age groups. The 4–10-year-old group did show a statistical significance before and after texting implementation (370 *µ*mol/l ± 99 versus 393 *µ*mol/l ± 129, respectively; *p*=0.021).

### 4.4. Retrospective Review of the Frequency (Number of Levels) of Phe Level Sampling before and after Introduction of Texting System (TS) (*µ*mol/l)

No statistical difference was found in the frequency of Phe levels before and after introduction of any group. The frequency of Phe refers to the number of levels over the period of 1 year. This is taking into account of the age range of the patient and frequency of levels which varies from weekly to monthly in patients over 2 years of age.

**Figure 1:**
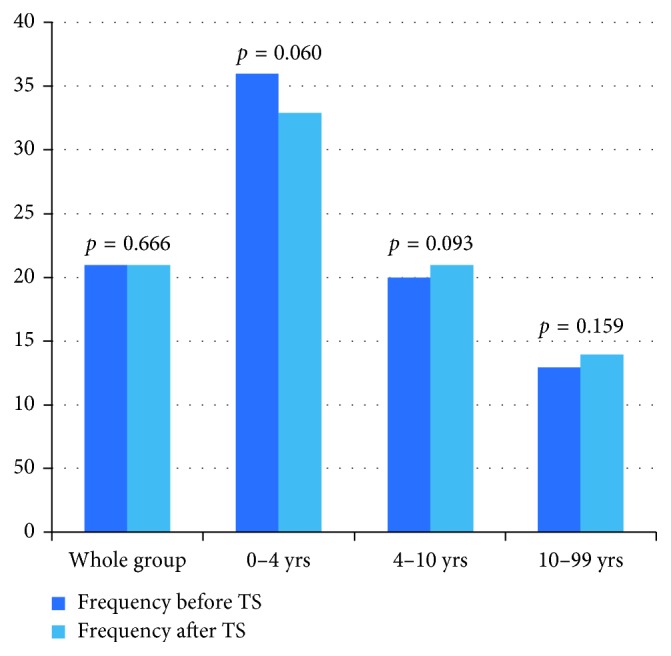


The percentage of texts within the normal range was 52%. This is exactly the same percentage as the initial audit carried out before the text system was implemented (Figure 1).

### 4.5. Survey Results January 2017

To date (August 2017), 290 eligible patients (61.5%) are signed up to the texting system. 16% (*N*=46) responded to the questionnaire. The main findings of the survey monkey were as follows:

- 87% found the system to be either very good or excellent.
- 71% found the system to have no impact on PKU blood levels.
- 84% felt that the system had no influence on dietary compliance.
- 94% found the system to be a time saver.

The complete survey monkey results are detailed in Tables 4–7.

### 4.6. Patient Comments

“I am all in favour of the texting. It is fast, efficient, and less time consuming. I think it is great. One can get the text no matter where they are.”

“Plus having the texting system reminds me of the levels as some weeks it has totally slipped my mind to ring. With the texting service that does not happen as you get the text either way.”

“I think texting is important as it saves time and also may be a reminder to parents to check levels.”

## 5. Discussion

The main objective of implementation of this system was as a quality improvement initiative in order to improve dietetic management in patients with PKU. Prior to the introduction of the texting system, the onus was on the patient to contact the NCIMD for results within a specified time period.

There was no detriment to the frequency or actual Phe levels after introduction of this system. The 4–10-year-old group did show a statistical significance before and after texting implementation, but this was not clinically significant.

In terms of the retrospective review of the frequency of Phe level sampling before and after introduction of the texting system, no statistical difference was found. The percentage of texts within the normal range was 52%.

This project required the integration of three disciplines in the hospital: Dietetics, ICT, and Metabolic Laboratory. The success of this project relied heavily on the collaboration and cooperation between these departments.

Feedback has confirmed that the system now in place is saving patients' time (Table 7). This frees up dietetic time for patients/parents who require more intense input when metabolic control is not stable and for new parents. A benefit of the system which was not thought of at the planning stage was that parents/patients now have a paperless record to refer to on their mobile. A study by Bilginsoy et al. in 2005 highlighted record keeping as one of the obstacles to better adherence in PKU [5]. Previously levels were recorded in books at home for reference.

Patient safety is always prioritised through the validation of results. A designated dietitian checks that all texts are sent and received once Phe results have been authorised by the laboratory. It is the parent's responsibility to contact the dietitian if levels are not within the range or the text message is not received. The text message sent is recorded on manual levels sheets, so there is a record if patients do not respond to out of range text messages. Unfortunately, there is no ICT system which flags these to the dietitian.

This project has huge potential to be applied in other therapeutic conditions which require regular blood test monitoring, for example, patients on warfarin or those with renal conditions. The main transferable feature of this project is the ability to link the laboratory system, for a variety of conditions, and the texting system by using technology. McGillicuddy et al. studied mobile phone-based programs for the kidney transplant recipient. They found a participation and retention rate of 41/55 (75%) and 31/34 (91%), respectively. This was a 3-month proof-of-concept randomized controlled trial which was conducted in 20 hypertensive kidney transplant patients [6]. Applebaum et al. carried out a survey in 20 patients aged 13–21 years from a paediatric university-based clinic. They identified a preference for using text messaging for communication in this cohort [7]. The use of text messages by a GP in London to inform patients about results of routine tests freed up greater than 600 appointments in the practice per year [8]. A study by Kerrison et al. in 2015 found that women who were sent a text message reminder before their first routine breast screening appointment were more likely to attend [9]. A study by Hunt in 2015 found that technology was a support to patients with diabetes in areas including taking medication and monitoring for complications [10].

The study period for the texting system was undertaken before the publication of the European Guidelines for Diagnosis and Management of PKU [11]. This study used the Irish reference range which suggests a range of 120–400 *µ*mol/l. In order to accurately analyse results, the original reference range was used for all analysis. From 1st July 2017, the ranges have been amended to the European guidelines for PKU [11].

The cost of implementing the system was minimal at €1000 for 2 licences. This project was not about saving money. However, savings of €3,275 which were a result of the reduced cost of a text versus a phone call for levels within the recommended range was an advantage in Ireland in recessionary times.

Patient satisfaction with the system was equally as important as patient safety. In order to assess the effectiveness of this quality improvement initiative, a patient survey was carried out in 2017. This showed 87% rated the system as either very good or excellent (Table 4). 94% agreed it was time saving which was very positive. 84% felt there was no influence on dietary compliance (Table 6). The system was not intended to impact dietary compliance in any way (Tables 3 and 5). Macdonald et al. cited technology as an aid in the reality of dietary compliance in PKU [12].

## 6. Conclusion

Text messaging Phe results is safe, time saving, and a more efficient use of dietetic time. This technology has the potential to be applied in other settings such as clinics and hospitals internationally.

## Figures and Tables

**Table 1:** Cost with and without use of the texting technology in euros.

	Normal Phe levels	Abnormal Phe levels	Total
Cost without texting (€)	3,484	3,225	6,709
Cost with texting (€)	209	3,225	3,434
Saving (€)			**3,275**

**Table 2:** Saving on dietetic hours.

	Number of levels	Time spent without texting (hours)	Time spent with texting (hours)
Normal Phe levels	3,484	None	**580.5**
Abnormal Phe levels	3,225	537.5	537.5
Total number of levels	6,709	537.5	1118

**Table 3:** Retrospective review of the mean Phe level before and after introduction.

	Mean before texting (±SD)	Mean after texting (±SD)	*p* value
Whole group	406 (±160) *µ*mol/l	416 (±188) *µ*mol/l	0.156
2–4 years	335 (±70) *µ*mol/l	326 (±79) *µ*mol/l	0.331
4–10 years	370 (±99) *µ*mol/l	393 (±129) *µ*mol/l	0.021
10+ years	483 (±207) *µ*mol/l	491 (±245) *µ*mol/l	0.555

**Table 4:** Overall how do you find the PKU texting system?

	Poor	Average	Good	Very good	Excellent
My rating of the texting system	0%	0%	13%	**34%**	**53%**

**Table 5:** Do you think the system has had an impact on you/your child's PKU blood levels?

	Disimproved	No change	Improved
PKU levels	0%	**71%**	**29%**

**Table 6:** Has the PKU texting system had an influence on you/your child in terms of dietary compliance?

	Better than before	Worse than before	No difference
Dietary compliance	16%	0%	**84%**

**Table 7:** Is the texting system saving you time?

	Yes	No
Saving you time	94%	6%
